# The Differences in Homocysteine Level between Obstructive Sleep Apnea Patients and Controls: A Meta-Analysis

**DOI:** 10.1371/journal.pone.0095794

**Published:** 2014-04-25

**Authors:** Xun Niu, Xiong Chen, Ying Xiao, Jiaqi Dong, Rui Zhang, Meixia Lu, Weijia Kong

**Affiliations:** 1 Department of Otolaryngology, Union Hospital, Tongji Medical College, Huazhong University of Science and Technology, Wuhan, China; 2 Department of Epidemiology and Biostatistics, and the Ministry of Education Key Lab of Environment and Health, School of Public Health, Tongji Medical College, Huazhong University of Science and Technology, Wuhan, Hubei, China; 3 Research Institute of Otorhinolaryngology, Tongji Medical College, Huazhong University of Science and Technology, Wuhan, Hubei, China; Idaho State University, United States of America

## Abstract

**Background:**

Studies have reported inconsistent findings regarding the relationship between obstructive sleep apnea (OSA) and homocysteine (HCY) level. This study aimed to assess the difference in plasma HCY level between OSA patients and controls by conducting a meta-analysis of published studies.

**Methods:**

Database of PubMed, SCI, and China National Knowledge Internet (CNKI) were comprehensively searched. Eligible studies regarding plasma HCY level in OSA patients were identified by two independent reviewers. RevMan (version 5.2) and STATA (version 12.0) were employed for data synthesis.

**Results:**

A total of 10 studies involving 432 subjects were included. Meta-analysis showed that plasma HCY levels in OSA group were 3.11 µmol/l higher than that in control group (95% confidence interval: 2.08 to 4.15, *P*<0.01). Subgroup analysis revealed a more significant differences between OSA patients and controls when average body mass index ≥30 (the total weighted mean difference (WMD) was 3.64), average age<50 (the total WMD was 3.96) and average apnea hypopnea index ≥35 (the total WMD was 4.54).

**Conclusions:**

In this meta-analysis, plasma HCY levels were found to be higher in OSA patients compared to control subjects.

## Introduction

Obstructive sleep apnea (OSA), characterized by repetitive hypopneas and apneas during sleep due to upper airway obstruction [1], [2], affects about 26% of the adults and has been gaining public awareness [3]. The relationship between OSA and cardiovascular conditions has been studied for decades, and OSA has been identified as an independent risk factor for cardiovascular diseases [4], [5]. However, the underlying mechanisms by which OSA causes cardiovascular complications were involved and have not yet been fully understood. Moreover, homocysteine (HCY) has been epidemiologically proved to be another independent risk factor for cardiovascular diseases [6]–[9], and to bear close relationship with their prognoses [6]. Elevated HCY was found to be associated with increased risk of coronary heart disease, hypertension and atherosclerosis [10].

If plasma HCY level in OSA patients is higher than their counterparts remain controversial. The findings of studies varied substantially, mainly as a result of small sample size or differences in study design. This study, by pooling the results of all relevant studies, tried to clarify whether plasma HCY level is elevated in OSA patients and to explore the possible involvement of OSA in cardiovascular complications.

## Methods

### Search Strategy

We searched for non-English and English articles included in SCI, PubMed and CNKI database. Search terms included the following key words: obstructive sleep apnea hypopnea syndrome, sleep apnea, obstructive sleep apnea, obstructive sleep hypopnea, sleep-disordered breathing, upper airway resistance and homocysteine. The computerized search was supplemented by a manual search of the bibliographies of all retrieved articles. Potentially relevant articles were evaluated for inclusion against pre-specified eligibility and exclusion criteria.

### Inclusion and exclusion criteria of literature

The studies were included if they satisfied the following criteria:

1. All subjects received monitoring by polysomnography (PSG); those whose apnea hypopnea index (AHI) ≥5 were assigned into case group, and those with apnea hypopnea index (AHI) <5 were included in control group.
2. All participants didn't take the medicines (such as methotrexate, folate, multivitamin, etc) that could affect experimental results.
3. No statistically significant difference was found between case group and control group in terms of age and body mass index (BMI).
4. All subjects were over 18 years.
5. All OSAHS patients were diagnosed for the first time, without receiving any form of treatment.
6. Plasma HCY concentration was measured from morning fasting venous blood.
7. The study provided sufficient data that allowed for a meta-analysis. A study was excluded if information available was not adequate for data extraction

Besides, abstract, letters to the editor and case reports were not included.

### Statistical Methods

Risk ratio (RR) and a 95% CI were used for presenting the statistical results for dichotomous outcomes. Weighted mean difference (WMD) and a 95% confidence interval (CI) were employed for presenting the statistical results for continuous outcomes. Mantel-Haenszel analysis was utilized for dichotomous variables and inverse variance method was used for continuous variables [11]. The statistical significance was set at *P*<0.05.

The difference was considered to be statistically significant if a *P* value was less than 0.10 and was also quantitatively assessed by using the value of I-square (I^2^<25%, no heterogeneity; I^2^ = 25–50%. moderate heterogeneity. I^2^ = 50–75%, moderate heterogeneity. I^2^>75%, high heterogeneity) [12]. If I^2^≤50%, showing that the studies were homogeneous or slightly heterogeneous, the fix effects model was used to combine the effect size. If I^2^>50%, indicating that the studies were moderately or highly heterogeneous, the random effects model was employed to combine the effect size [13], [14]. Statistical calculations were performed by using STATA version 12.0 and Review Manager 5.2.

Subgroup analysis was performed to access the impact of age (<50 and ≥50), BMI (<30 and ≥30) and AHI (<30 and ≥30). Sensitivity analysis was used to evaluate the stability of the result of the meta-analysis. We conducted meta-regression to identify the possible sources of heterogeneity. Forest plot was computer-generated. Potential publication bias was assessed by using funnel plot [15], the Begg test and the test of Egger *etc* [15], [16]. We also employed trim and fill method to identify and correct for funnel plot asymmetry arising from publication bias [17].

## Results

### Search Results

**Figure 1:**
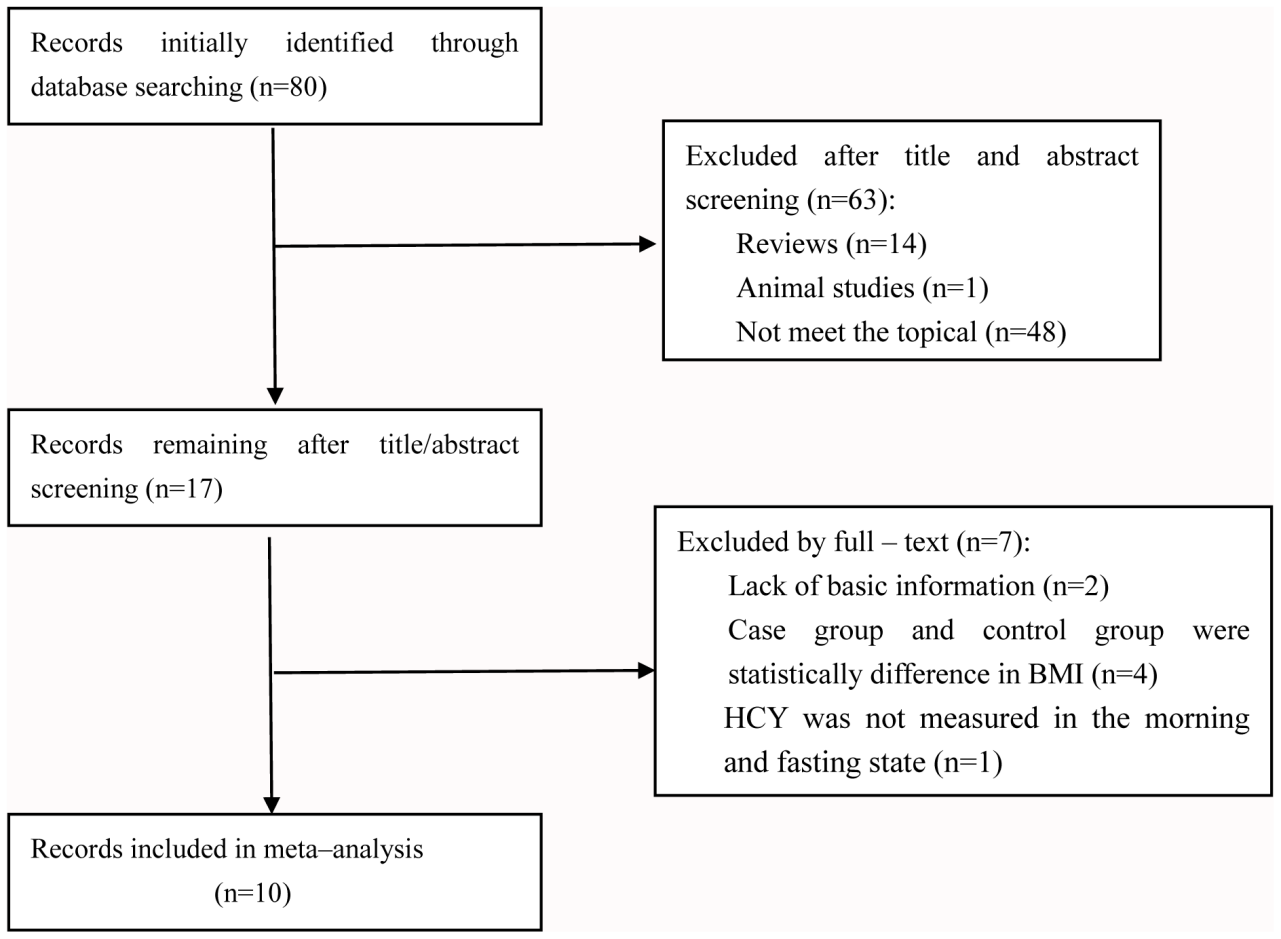
Flow chart of the study selection process. After careful discussion between the 2 reviewers, a total of 10 studies were included to perform the meta-analysis.

The initial search was independently executed by two reviewers, and 80 articles were preliminarily selected. Screening by title and abstract was conducted in accordance with inclusion/exclusion criteria. After a thorough discussion between the 2 reviewers, 17 articles were found to be related to this study. The 17 articles then were subjected to second-stage review. Finally, a total of 10 studies were included for the meta- analysis. The detailed steps of the literature search are shown in **Figure 1**.

### Characteristics of the Eligible Studies

Ten studies [18]–[27], covering data from a total of 432 participants, were included in this review. The information of authors, publication year, national sources, sample size and the level of evidence of each study were listed in **Table 1**. All the included case-control trials are defined as level 3 and cross-sectional trails are defined as level 2, basing on the study design [28]. The information of mean age, BMI, AHI and HCY of each study are given in **Table 2**.

**Table 1:** Characteristics of included studies.

Author	Year	country	Study design	LOE	Sample Size (OG/CG)
Lavie L *et al.* [18]	2001	Israel	CCT	3b	49/35
Kokturk O *et al.* [19]	2006	Turkey	CST	2b	25/42
Yesim Ozkan *et al.* [20]	2008	Turkey	CST	2b	34/15
Wang xia *et al.* [21]	2008	China	CST	2b	81/120
Wang ling *et al.* [22]	2010	China	CST	2b	32/29
Fatima Cintra *et al.* [23]	2011	Brazil	CCT	3b	14/20
Chen M *et al.* [24]	2011	China	CST	2b	102/27
Basoglu OK *et al.* [25]	2011	Turkey	CST	2b	36/34
Monneret D *et al.* [26]	2012	France	CST	2b	26/9
Sales LV *et al.* [27]	2013	Brazil	CST	2b	14/13

Abbreviations: **CCT**, case-control trial; **CST**, cross-sectional trail; **LOE**, level of evidence; **3b**, level 3; **2b**, level 2; **OG**, obstructive sleep apnea-hypopnea syndrome group; **CG**, control group.

**Table 2:** Characteristics of included studies.

Author	Mean(SD) HCY,µmol/l		Mean BMI		Mean Age,y		Mean AHI,Events/h	
	OG	CG	OG	CG	OG	CG	OG	CG
Lavie L *et al.* [18]	14.6(6.7)	11.9(5.8)	30.2	28.7	60.1	60.0	30.2	<5
Kokturk O *et al.* [19]	17.2(6.6)	10.4(3.6)	32.3	27.2	51.8	48.9	44.9	2.1
Yesim Ozkan *et al.* [20]	16.4(5.7)	11.2(5.9)	30.8	27.4	48.7	43.5	39.3	1.3
Wang xia *et al.* [21]	14.0(2.9)	14.5(2.3)	25.5	25.1	52.6	50.2	>5	<5
Wang ling *et al.* [22]	10.8(2.6)	8.9(1.2)	28.4	25.1	42.7	44.7	45.5	3.4
Fatima Cintra *et al.* [23]	16.5(4.7)	15.1(3.6)	23.1	22.5	53.4	53.1	18.1	<5
Chen M *et al.* [24]	13.3(5.4)	9.0(3.7)	24.8	22.0	58.7	58.8	62.2	2.8
Basoglu OK *et al.* [25]	18.1(2.7)	17.9(4.5)	33.5	34.5	50.0	50.1	27.2	<5
Monneret D *et al.* [26]	12.8(3.8)	9.5(2.5)	29.8	29.1	61.5	59.7	31.7	3.4
Sales LV *et al.* [27]	16.7(8.0)	10.7(2.9)	28.8	26.9	37.2	36.0	36.4	1.9

Abbreviations: **OG**, obstructive sleep apnea-hypopnea syndrome group; **CG**, control group; **SD**, standard deviation; **HCY**, homocysteine; **BMI**, body mass index; **AHI**, apnea - hypopnea index.

### Pooled Analysis

**Figure 2:**
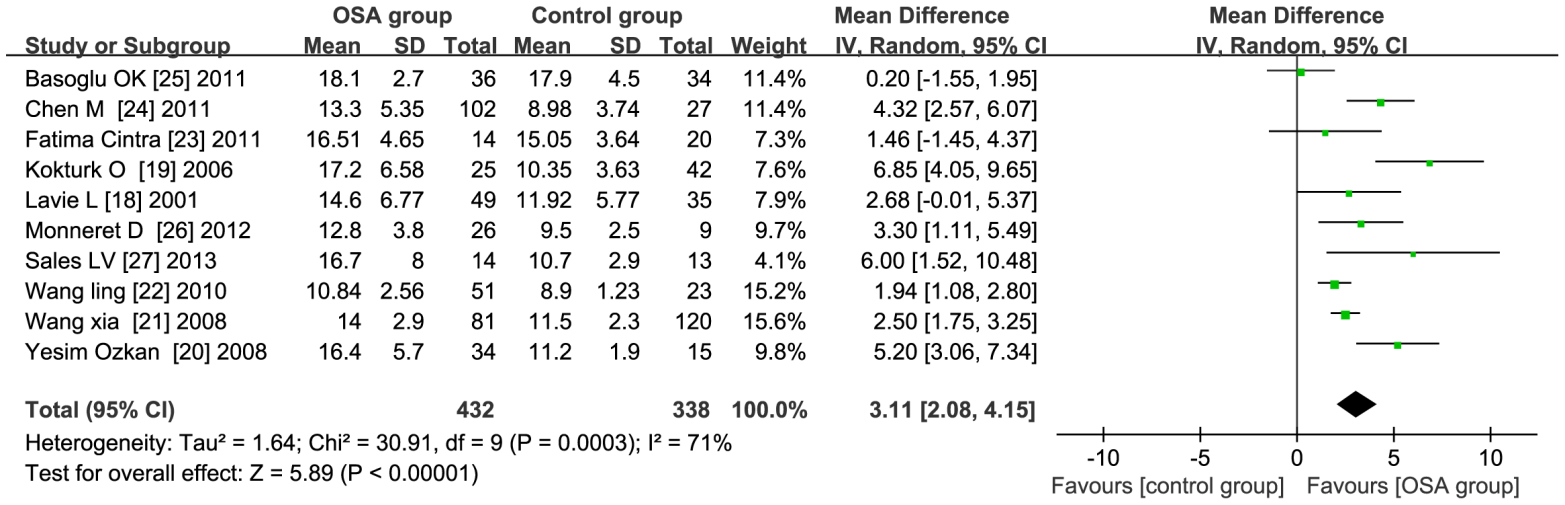
Comparison of honocysteine levels between OSA group and control group in the 10 included studies. Calculation based on random effects model. Results are expressed as weighted mean difference (WMD) and 95% confidence intervals (95% CI). Meta-analysis showed that the total WMD for the homocysteine levels was 3.11 µmol/l. AS such, if all studies are included, homocysteine levels were found to be higher in OSA patients compared to control subjects.

The value of I^2^ was 71%, indicating that the studies were moderate heterogeneous. Therefore, the random effects model was used to combine effect size. Meta-analysis exhibited that plasma HCY levels in OSAHS group were 3.11 µmol/l higher than that in control group (95% CI: 95% CI: 2.08 to 4.15, *P*<0.01) **Figure 2**.

### Subgroup Analysis - population type

**Figure 3:**
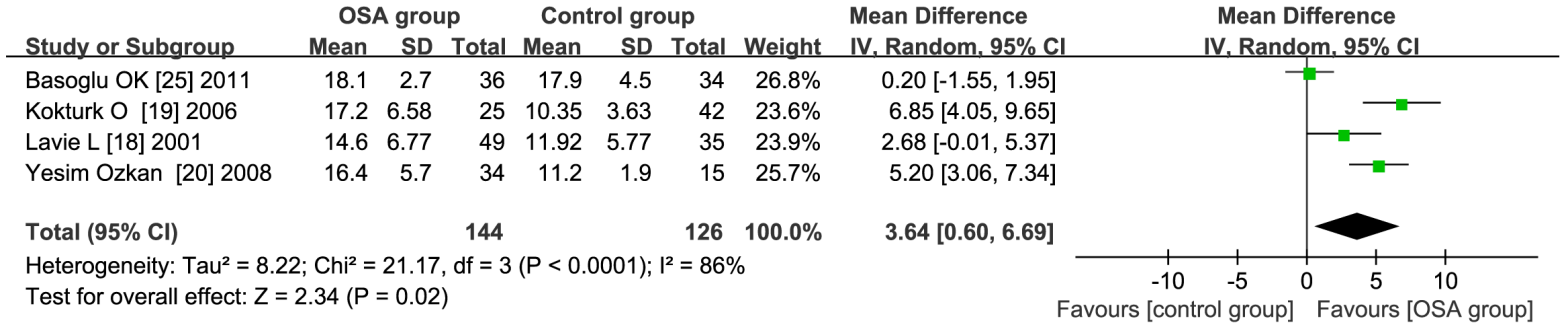
Subgroup analysis based on BMI≥30. Calculation based on random effects model. Results are expressed as weighted mean difference (WMD) and 95% confidence intervals (95% CI). The total WMD in the studies with average BMI≥30 is significant, with a corresponding value of 3.64(95% CI: 0.60 to 6.69, *P*<0.05).

**Figure 4:**
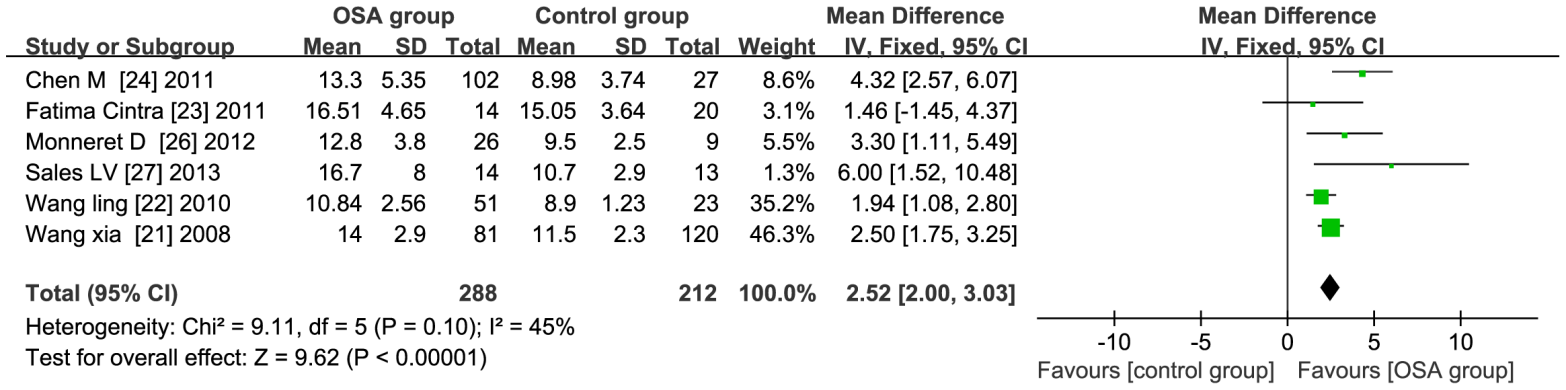
Subgroup analysis based on BMI<30. Calculation based on fix effects model. Results are expressed as weighted mean difference (WMD) and 95% confidence intervals (95% CI). The total WMD in the studies with average BMI<30 is significant, with a corresponding value of was 2.52 (95% CI: 2.00 to 3.03, *P*<0.01).

**BMI≥30**: The total WMD in the studies with average BMI≥30 was 3.64 (95% CI: 0.60 to 6.69, *P*<0.01) **Figure 3**. **BMI<30** the total WMD in the studies with average BMI<30 was 2.52 (95% CI: 2.00 to 3.03, *P*<0.01) **Figure 4**.

### Subgroup Analysis -Age

**Figure 5:**
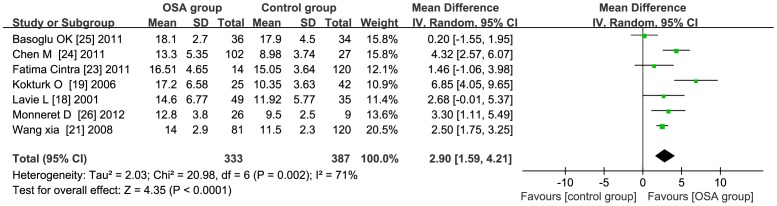
Subgroup analysis based on average age beyond 50. Calculation based on random effects model. Results are expressed as weighted mean difference (WMD) and 95% confidence intervals (95% CI). The total WMD in the studies with average age beyond 50 is significant, with a corresponding value of 2.90 (95% CI: 1.59 to 4.21, *P*<0.01).

**Figure 6:**
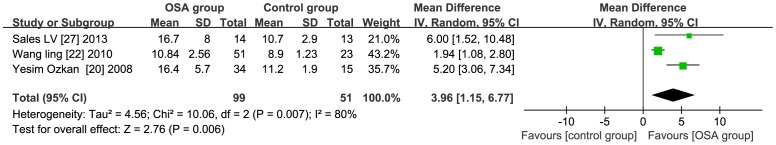
Subgroup analysis based on average age less than 50. Calculation based on random effects model. Results are expressed as weighted mean difference (WMD) and 95% confidence intervals (95% CI). The total WMD in the studies with average age less than 50 is significant, with a corresponding value of 3.96 (95% CI: 1.15 to 6.77, *P*<0.01).

**Age≥50** the total WMD in the studies with average age beyond 50 was significant, with a corresponding value of 2.90 (95% CI: 1.59 to 4.21, *P*<0.01) **Figure 5**. **Age<50** the total WMD in the studies with average age less than 50 was significant, with a corresponding value of 3.96 (95% CI: 1.15 to 6.77, *P*<0.01) **Figure 6**.

### Subgroup Analysis-AHI

**Figure 7:**
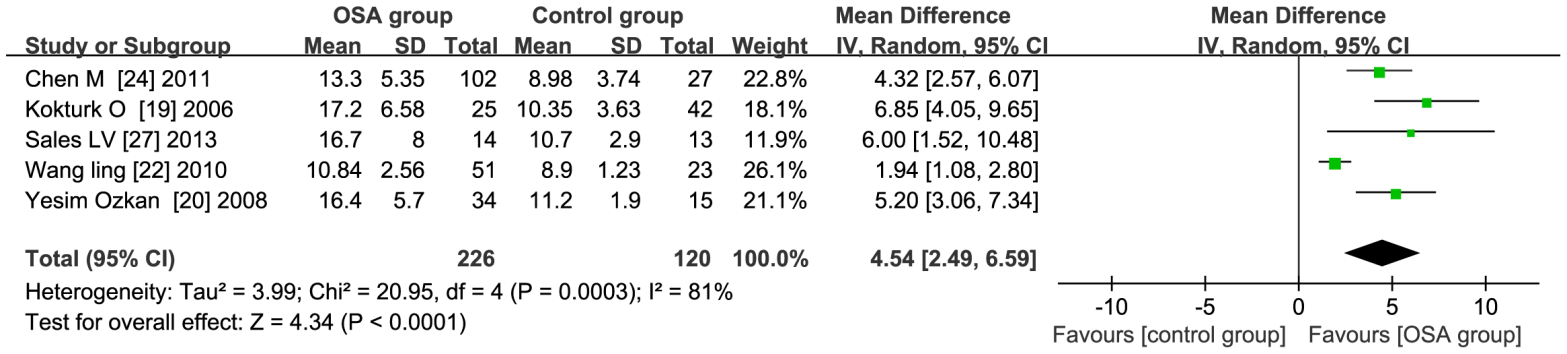
Subgroup analysis based on average AHI≥35. Calculation based on random effects model. Results are expressed as weighted mean difference (WMD) and 95% confidence intervals (95% CI). The total WMD in the studies with average AHI≥35 is significant, with a corresponding value of 4.54 (95% CI: 2.49 to 6.59, *P*<0.01).

**Figure 8:**
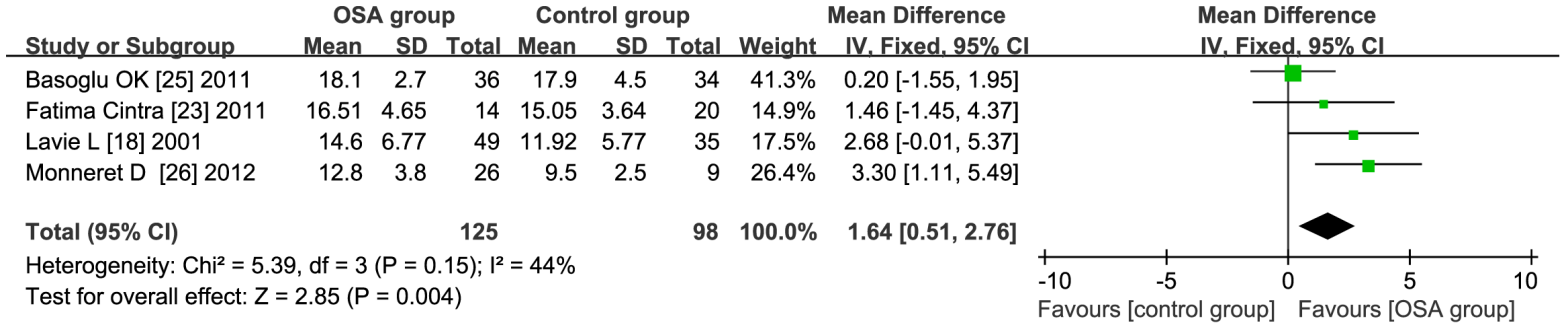
Subgroup analysis based on average AHI<35. Calculation based on fix effects model. Results are expressed as weighted mean difference (WMD) and 95% confidence intervals (95% CI). The total WMD in the studies with average AHI<35 is significant, with a corresponding value of 1.64 (95% CI: 0.51 to 2.76, *P*<0.01).

**AHI≥35** the total WMD in the studies with average AHI≥35 was significant, with a corresponding value of 4.54 (95% CI: 2.49 to 6.59, *P*<0.01) **Figure 7**. **AHI<35** the total WMD in the studies with average AHI<35 was significant, with a corresponding value of 1.64 (95% CI: 0.51 to 2.76, *P*<0.01) **Figure 8**.

### Sensitivity Analysis

Sensitivity analysis showed that removal of any study from the analysis did not subvert the present pooled analysis result (data not shown). After including the previously excluded studies [29], [30], which failed to mention if their subjects took any drug that could affect the result of their experiments, the pooled analysis result was 3.29 (95% CI: 2.07 to 4.52, *P*<0.01). Pooled analysis using random-effects model showed that HCY levels were increased significantly (WMD: 3.11, 95% CI: 2.08 to 4.15,*P*<0.01). The fixed-effects model draw a similar result (WMD:2.60, 95% CI: 2.14 to 3.07, *P*<0.01).

### Publication Bias

**Figure 9:**
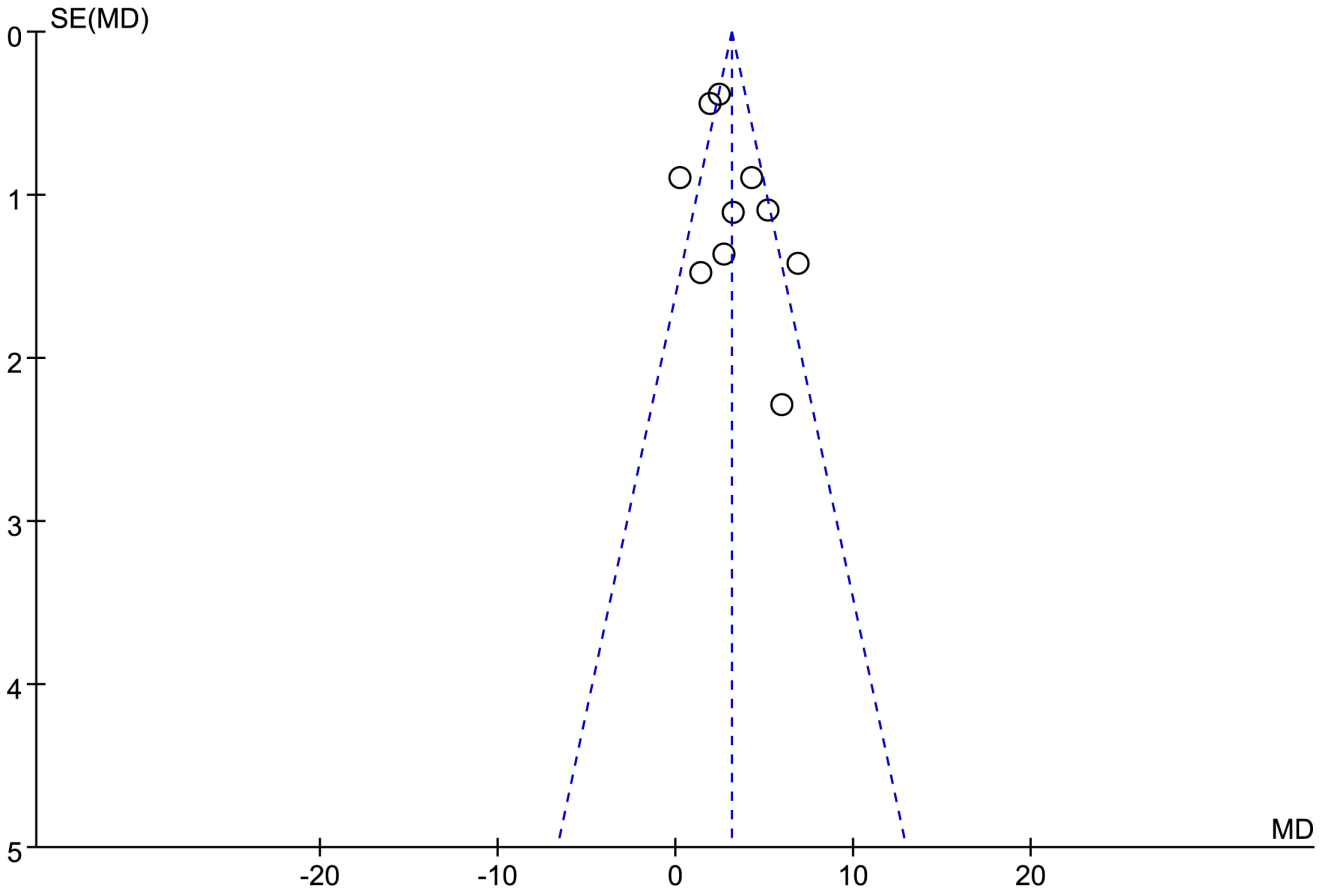
Funnel plot showed the possibility of a small publication bias. SE, standard error, MD, mean difference.

The funnel plot was not perfectly symmetrical (**Figure 9**), suggesting that there might be slight publication bias, but Begg tests (*P* = 0.65) and Egger tests (*P* = 0.22) didn't provide evidence of publication bias in our study. In addition, the trim and fill method showed that no study needed to be statistically corrected for funnel plot asymmetry.

### Meta-regression analysis

In univariate meta-regression analysis, the outcome variable was the WMD of HCY level and the covariates included average age, BMI, AHI, publication year and race that might influence the outcome. The HCY levels were not significantly correlated with the age of patients (*P* = 0.55), BMI of patients (*P* = 0.07), age of normal participants (*P* = 0.80), BMI of normal participants (*P* = 0.07), publication year (*P* = 0.532), race (*P* = 0.441) and AHI (*P* = 0.90).

## Discussion

HCY has been recently identified as an independent risk factor for cardiovascular diseases [31] and, pathophysiologically, it promotes cardiovascular events by inducing endothelial dysfunction and coagulation abnormalities [32]. Bouchey et al found that for each increase in plasma HCY by 5 µmol/l, the risk of coronary disease increased by 60%∼80% and the incidence of cerebrovascular diseases increased by 50% [33]. A study by John W *et al* suggested that hyperhomocysteine (HHCY) might account for 10% in the total risk of cardiovascular diseases, and the risk of cardiovascular events was lowered by about 25% if the level of HCY was reduced [34]. Similarly, some researchers found that OSA was also an independent risk factor for cardiovascular diseases [35], [36] and more than half of the patients with cerebrovascular diseases had OSA [37], [38].

The current study utilized meta-analytic methods to evaluate the difference in plasma HCY levels between OSA patients and control group. Our results indicated that plasma HCY levels were significantly raised in OSA patients. Moreover, in sensitivity analysis, after any study was removed, fixed-effects model convert to random effects model or inclusion/exclusion criteria were changed, the overall results and conclusion were not affected. Therefore, the outcome of our meta-analysis could be regarded with a higher degree of certainty.

To further understand whether BMI, age and AHI would have impact on plasma HCY levels, we performed subgroup analysis in terms of BMI, age and AHI. The results showed that the parameters had a more significant effect on HCY levels when average BMI≥30, age<50 and AHI≥35. However, although in each individual study, difference in baseline BMI was not significant between patients and controls, our results suggested that there existed a more significant difference in plasma HCY levels between obese OSA patients and their obese controls without OSA. Similarly, although no significant difference was found in mean age between the patients and controls, our analysis suggested that plasma HCY levels in the younger OSA patients may increase more significantly than their controls. In addition, our analysis suggested that plasma HCY levels in the severe OSA patients might increase more significantly and the finding was consistent with the studies [24], [27].

Despite these meaningful findings, our study was not without limitations. First, the meta-analysis included 3 case-control trials and 7 cross-sectional trails, each, possibly, having a degree of experimental bias. Second, the number and size of studies included in the analysis was relatively small. The larger and more numerous studies would allow for more precise effect size estimation as well as more sophisticated moderator analysis. Third, a moderate heterogeneity was present among individual studies, but we failed to find exact source of heterogeneity from the limited studies included.

## Conclusion

Although there are still some debates concerning the relationship between OSA and HCY, our analysis suggested that plasma HCY levels in OSA patients were 3.11 ummol/L higher than controls. Therefore, we were led to speculate that the elevated HCY might be one of the mechanisms responsible for OSA-related cardiovascular complications. Whether plasma HCY can be used as an indicator of the risk of cardiovascular diseases for OSA patients and the possibility of delaying or preventing cardiovascular diseases by reducing plasma HCY level in OSA patients warrant further study.

## Supporting Information

Checklist S1 PRISMA Checklist of this meta-analysis.
(DOC)
